# A hybrid type I randomized effectiveness-implementation trial of patient navigation to improve access to services for children with autism spectrum disorder

**DOI:** 10.1186/s12888-018-1661-7

**Published:** 2018-03-27

**Authors:** Sarabeth Broder-Fingert, Morgan Walls, Marilyn Augustyn, Rinad Beidas, David Mandell, Shannon Wiltsey-Stirman, Michael Silverstein, Emily Feinberg

**Affiliations:** 1 Department of Pediatrics, Boston University School of Medicine, Boston, MA 02114 USA; 2 Division of Developmental and Behavioral Pediatrics, Boston University School of Medicine, Boston, MA 02114 USA; 3 Center for Mental Health Policy and Services Research, Perelman School of Medicine, University of Pennsylvania, Philadelphia, PA 19104 USA; 4 National Center for PTSD, Dissemination and Training Division, Menlo Park, CA 94025 USA; 5 Department of Community Health Sciences, Boston University School of Public Health, Boston, MA 02114 USA; 6 Division of General Pediatrics, Boston University School of Medicine, 850 Harrison Ave, Room 310A, Boston, MA 02118 USA

**Keywords:** Autism spectrum disorder, Navigation, Disparities

## Abstract

**Background:**

Significant racial, ethnic, and socioeconomic disparities exist in access to evidence-based treatment services for children with autism spectrum disorder (ASD). Patient Navigation (PN) is a theory-based care management strategy designed to reduce disparities in access to care. The purpose of this study is to test the effectiveness of PN a strategy to reduce disparities in access to evidence-based services for vulnerable children with ASD, as well as to explore factors that impact implementation.

**Methods:**

This study uses a hybrid type I randomized effectiveness/implementation design to test effectiveness and collect data on implementation concurrently. It is a two-arm comparative effectiveness trial with a target of 125 participants per arm. Participants are families of children age 15–27 months who receive a positive screen for ASD at a primary care visit at urban clinics in Massachusetts (*n* = 6 clinics), Connecticut (*n* = 1), and Pennsylvania (*n* = 2). The trial measures diagnostic interval (number of days from positive screen to diagnostic determination) and time to receipt of evidence-based ASD services/recommended services (number of days from date of diagnosis to receipt of services) in those with PN compared to and activated control -Conventional Care Management – which is similar to care management received in a high quality medical home. At the same time, a mixed-method implementation evaluation is being carried out.

**Discussion:**

This study will examine the effectiveness of PN to reduce the time to and receipt of evidence-based services for vulnerable children with ASD, as well as factors that influence implementation. Findings will tell us both if PN is an effective approach for improving access to evidence-based care for children with ASD, and inform future strategies for dissemination.

**Trial registration:**

NCT02359084 Registered February 1, 2015.

## Background

### Evidence-based services for autism spectrum disorder

Over 15 years of data support the notion that earlier access to evidence-based services (EBS) improves both short and long-term outcomes for children with autism spectrum disorder (ASD) [1]. Studies consistently demonstrate that intensive early intervention improves cognition, language skills, and reduces the core symptoms of ASD [2–4], and that earlier access to these services leads to both immediate benefits, and gains over time [5].

### Disparities in autism services

Significant disparities exist in access to EBS for children with ASD. On average, low-income and minority children with ASD are diagnosed later than their white and higher income counterparts, and they experience substantial delays in initiating treatment – even after diagnosis [6, 7]. Because earlier access to evidence-based ASD services improves both short and long-term outcomes, delayed engagement with these services can be responsible for substantial morbidity [8]. Obtaining an ASD diagnosis and engaging with treatment involves a number of complex steps, including visits with a primary care provider for screening, visits with a subspecialist for diagnosis, receipt of an individually-tailored treatment plan, and ongoing early intervention and special education support [9]. Barriers to timely ASD diagnostic and treatment services can result from a variety of factors including the availability of services, patient-provider miscommunication, parental stress, complex payment systems, and culturally biased care [10–12].

### Patient navigation

Patient Navigation (PN) is a theory-based and empirically-supported care management strategy designed to reduce disparities in access to care, and thus represents a promising strategy to help low-income and minority families access timely evidence-based ASD services. PN focuses on overcoming patient-specific barriers to a defined set of services over a time-limited period. It is rooted in the Chronic Care Model [13], and has empiric evidence in diseases such as cancer and HIV as a means to reduce disparities in outcomes [14–16] by shortening the interval between a positive screening test (for example, a mammogram for breast cancer) and definitive diagnosis. Early pilot data demonstrate that PN has the potential to reduce the time from initial ASD screening to diagnosis, and to improve access and retention in treatment services [17].

### Rationale for hybrid design

Despite the promise of PN as a stretgy to reduce disparities in access to EBS for this population, multiple studies show that access to new innovations, particularly in mental health, is often significantly delayed for low-income and minority populations [18]. This gap in equitable access to evidence-based care has led leaders in healthcare policy to call for research that specifically addresses the adoption and spread of innovations – like PN – that promote engagement with treatment services for vulnerable patients [19]. Consistent with this concern, multiple studies in cancer and HIV demonstrate varying success or diminution of impact of PN upon implementation in real-world practice [20–22]. Therefore, the current protocol describes a hybrid implementation-effectiveness trial in which we will evaluate both the effectiveness of PN, while at the same time assessing factors that influence its implementation. The purpose of this trial design is to prepare for the rapid dissemination of PN as a strategy to reduce disparities in ASD engagement in EBS, if proven effective.

## Methods/design

### Overview

**Fig. 1:**
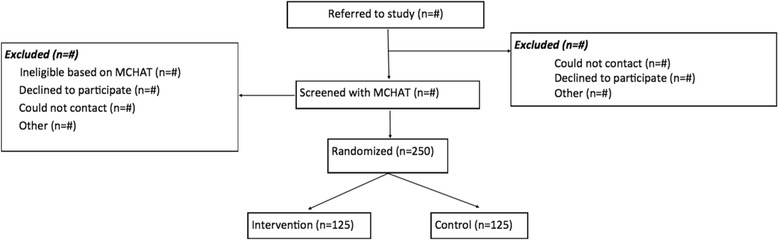
CONSORT diagram

This study compares PN to Conventional Care Management (CCM) on two primary outcomes - diagnostic interval and time to receipt of evidence-based ASD/recommended services. A number of secondary outcomes such as number of children diagnosed with ASD, satisfaction with the navigator, and an array of parent-reported measures (e.g. parenting stress, social support, and coping responses) will be evaluated as well. Using a mixed methods approach (process mapping, qualitative, and quantitative data analyses), we will also assess implementation of PN, with a focus on identifying failures in implementation, why they exist, and how to mitigate them for future adoption and spread. Our study is designed as a type I hybrid effectiveness/implementation evaluation to provide the evidence needed for patients, clinicians, and policy makers to determine if PN should be offered to children at risk for ASD in primary care, and how it can be spread to clinical practice after the trial is complete. The study protocol described received approval from the Boston University Institutional Review Board (protocol number H-33008). See Fig. 1 for full CONSORT diagram.

### Conceptual framework

Our intervention is informed by the Chronic Care Model, which identifies six fundamental areas that form a system that encourages high-quality chronic disease management (self-management support, delivery system design, decision support, clinical information systems, organization of health care, and community) [23]. The model was developed to help systems create supportive interactions between an informed, activated patient and a prepared, proactive practice team. Our navigation strategy uses the following principles of the Chronic Care Model: 1) navigators are trained in community and health system resources for ASD to allow for integration of services across sectors; 2) navigators are trained in motivational interviewing and collaborative decision making to support proactive interactions between patients and providers. Our implementation evaluation is guided by the Consolidated Framework for Implementation Research (CFIR) developed by Damschroder and colleagues [24], and preliminary data from Drahota and colleagues’ Autism Model of Implementation (AMI) [25], which also draws from CFIR. We will assess PN across all five domains of CFIR (Intervention Characteristics, Outer setting, Inner setting, Characteristics of Individuals, Process), by exploring multiple constructs from each domain. We chose CFIR because it has two specific advantages over other determinant frameworks that apply to the current project: 1) CFIR offers an overarching typology to promote theory development and verification about what works where and why across multiple contexts. Therefore it is most appropriate for formative work, in which specific causal mechanisms for implementation success are not hypothesized a priori; 2) CFIR contains a broad range of contextual dimension (5 domains and 26 discrete constructs) that describe the internal and external context of implementation. Therefore it is particularly suitable for studying implementation in multiple settings and diverse populations.

### Setting

The setting for this hybrid type I randomized effectiveness/implementation study is three urban primary care networks affiliated with Boston Medical Center (BMC), Children’s Hospital of Philadelphia (CHOP), and Yale University (Yale), and their Developmental and Behavioral Pediatrics (DBP) autism specialty clinics. Each DBP clinic is a member of the HRSA-funded DBPNet – a 12-site practice-based research network whose mission is to conduct collaborative, interdisciplinary research in developmental and behavioral pediatric settings. Clinical sites include three types of primary care practices – hospital, community, and health center – and are located in three states (Massachusetts, Connecticut, and Pennsylvania) with different requirements for accessing ASD services. These sites serve a diverse population of over 7000 children in the target age range each year.

### Participants

Because our study is designed to test a strategy to reduce disparities, our recruitment efforts focus on urban, racial and ethnically diverse populations - who historically report low levels of engagement with the healthcare system - within our three study sites. Participants will be children 15 to 27 months with a positive The Modified Checklist for Autism in Toddlers, Revised with Follow-Up (MCHAT-R/F) screen at their health supervision visit, or those who are referred by their pediatrician based on clinical concern. The MCHAT-R/F is a 2-stage parent-report screening tool to assess risk for ASD. The initial screen is performed in the primary care pediatrician’s office and consists of 20 yes/no questions. The scores range from “low risk” (0–2), “medium risk” (3–7), or “high risk” (8–20). Children who score “high risk” or “medium risk” are referred to our study by the primary care physician. Children who are “high risk” are screened for enrollment into the study. Children who are “medium risk” are administered a follow up interview (per MCHAT-R/F protocol) by study staff, and are referred to the study if positive. Because this is designed as a pragmatic trial, inclusion criteria are broad. Specific inclusion criteria are as follows: 1) Age 15–27 months at time of referral; and 2) MCHAT-R/F score demonstrating risk for ASD or provider concerns for ASD. The only exclusion criteria is a prior diagnosis of ASD. There are no exclusions based on language spoken or co-morbid condition.

### Control and intervention groups

#### Control

The control arm of this study receives Conventional Care Management consistent with the type of care provided within a traditional - but high quality – medical home. The care manager is available by phone to provide families with phone numbers and resources related to parents’ concerns about their child’s appointment, diagnosis, or developmental services.

The care manager contacts families by phone after randomization to introduce herself and remind families of their first appointment at the developmental clinic for their child’s ASD evaluation. The child’s primary care clinician also receives a letter from the care manager informing the clinician that the family has been assigned a care manager. The letter also contains the care manager’s contact information. After the care manager’s introductory phone call and letter, she remains available to the child’s family and other members of the care team if and when they initiate contact.

### Intervention

PN is a theory-based, empirically-supported, multicomponent, manualized care management strategy [26, 27]. It differs from other care management strategies in that it focuses on overcoming patient-specific barriers to a defined set of services over a defined, time-limited period. PN is well established in cancer care as a means to reduce disparities in the critical interval between a positive screening test (for example, a mammogram for breast cancer) and definitive diagnosis [16, 17, 28], decrease anxiety [29], and increase satisfaction with services [29]. The goal of PN is to integrate a disjointed health care system on behalf of an individual patient. In our proposal, we build upon the established principles of PN but expand the navigator role in novel ways. Navigation services are provided to the family unit by bilingual paraprofessionals. It is extended to integrate a fragmented network of ASD services that requires coordination among community-based and educational services, as well as those provided by the conventional health care team.

Additionally, PN is augmented with evidence-based behavior change strategies – motivational interviewing and collaborative decision making – that support patient engagement and self-management skills. A typical PN activity may be to assist a parent with marginal literacy to complete a clinic’s patient questionnaires, help a family know what to expect during a stressful ASD diagnostic evaluation, or coordinate childcare around a child’s early intervention schedule. In this proposal, the systemic PN protocol begins after referral for an autism evaluation and end 100 days after diagnostic resolution – at which time, a child with ASD would be expected to be engaged and retained in intensive early intervention services (based on the guidance from Autism Speaks) [30]. Navigators follow a standardized protocol, keep extensive structured logs of their work, and a random subset of encounters are audio recorded. Data are captured on all interactions with families, providers, medical, educational, and social service organizations, recording time spent, activities performed, and barriers addressed.

### Randomization process

We use stratified, blocked (randomly varying blocks of 2 and 4) randomization to allocate participants to Navigation or CCM. Randomization occurs independently at each primary care recruitment site. Randomization lists are generated in a secure web-based data management system. All outcome assessors are blinded to participant allocation. Written and verbal informed consent is obtained by research staff prior to randomization. Data will be collected in stored in StudyTrax, a commercial clinical research data management tool.

### Effectiveness outcomes

#### Primary outcomes

The study’s primary outcomes are access to screening, diagnostic, and evidence-based service outcomes (Table 1). The trial measures diagnostic interval (number of days from positive screen to diagnostic determination) and time to receipt of ASD services/recommended services (number of days from date of diagnosis to receipt of services) from review of child’s medical and early intervention records in those with PN compared to CCM.

**Table 1:** Diagnostic and Service Outcomes (primary outcomes)

Outcome	Measures	Response	Description
Expedited Evaluation	Achievement of Diagnostic Resolution	Yes/No	Percent children who complete diagnostic assessment within 12-month follow-up
	Timing of Diagnostic Resolution	Timely	Timely- Diagnostic interval ≤ 120 days
		Delayed	Delayed- Diagnostic interval > 120 but ≤ 180 days
		Unresolved	Unresolved - No resolution by 12-months
	Diagnostic Interval	Number of Days	Beginning the day of the positive confirmatory screen and ending the day when family receives determination (yes/no) of ASD diagnosis
Referral to Treatment	Time to receipt of EI services	Number of Days	Days from failed M-CHAT to receipt of services
	Time to receipt of ASD services	Number of Days	Days from Date of diagnosis to ASD services
Engagement in Treatment	Hours of services	Number of Hours	Hours ASD specialty services
	Adequacy of Services	Yes/No	Based on guideline of 25 h/ week
	Absenteeism	Number of absences	Number of family-initiated “no show” and cancellations divided by the number of scheduled appointments in 6 month period following diagnosis
Determination of ASD Diagnosis	ASD diagnosis	Yes/No	Based on ADOS evaluation by Board Certified DBP
Satisfaction with ASD-Services	Client Satisfaction Questionnaire [50]	Categorical and numerical	Previously validated measure of satisfaction with services
	Satisfaction with Hospital Care Questionnaire [50]	Categorical and numerical	Previously validated measure of satisfaction with services
Satisfaction with Navigator	Patient Satisfaction with Interpersonal Relationship with Navigator [51]	Categorical and numerical	Newly validated 9-item scale to assess satisfaction with the interpersonal relationship with navigators

### Secondary outcomes

A number of secondary outcomes such as number of children diagnosed with ASD, satisfaction with the navigator, and an array of parent-reported measures (e.g. parenting stress, social support, and coping responses) and are assessed (see Table 1 for full list of measures) at the time of entry into the study, as well as at three follow-up time points. Additional process outcomes will include confirmatory screening results, referrals to care, and service use and are obtained from care manager and navigator logs and from the child’s medical, early intervention, and ASD service provider records.

### Analyses

#### Intervention main effects

We will conduct an intention-to-treat (ITT) analysis where the denominator will include all children randomized to a treatment arm. For categorical outcomes we will use logistic regression models to compare the proportion of children who completed the diagnostic evaluation, and received recommended ASD specific services.

For time-to-event outcomes (time to diagnostic resolution, and time to receipt of ASD services) we will use censored analyses to construct Kaplan-Meier curves of time to event, and estimate hazard ratios, using Cox proportional hazard models.

### Mediator/moderator analyses

#### Moderation analyses

We have identified two a priori, theory-based potential effect modifiers: child race/ethnicity and site. We will perform stratified analyses to identify the nature of such moderation, followed by formal testing of interaction terms in our statistical models.

#### Center effects

One of the strengths of our study is that each clinic site (center) is unique – in terms of its own clinical processes and the early intervention sites it has access to. By randomizing our sample separately within each center, we eliminate the potential of confounding by center. However, we will also examine potential clustering and assess effect modification by center [31], which will help us determine the generalizability of the PN model.

#### Racial and ethnic group effects

Specific barriers to ASD identification and service provision differ by race and ethnicity [32–35]. A strength of our sample is that we can examine the impact of the intervention for key population subgroups that have been under-screened, −assessed, and -treated for ASD.

#### Examination of intervention mechanism

Given our intervention’s emphasis on goal setting and action planning, decreased caregiver burden, decreased perceptions of stress, and behavioral activation constitute likely intervention mediators. We will examine mediational effects using two different, but related, methods: the approach of Baron and Kenny [36] and the use of path analysis models. [37] Each of these approaches can be used to differentiate between direct and indirect intervention effects. In the path analysis models, we will compare the fit of meditational vs. non-mediational models by differences in Akaike’s Information Criterion, the comparative fit index (optimal value > 0.95), the Tucker-Lewis index (optimal value > 0.95), and the root mean square error of approximation (optimal values < 0.06). We will fit these models with MPlus software, which allows for the modeling of measurement and dichotomous, endogenous and exogenous variables.

### Sample size and power

According to Kraemer’s threshold of clinical significance concept [38], we estimate a required sample size of 250 to detect the smallest differences in primary outcomes that are of clinical importance. In the ITT analysis, we assess the adequacy of the expected sample to detect main effects among all children randomized, assuming a two-sided alpha of 0.05. We present power calculations for planned categorical analyses. Power to detect between group differences in analyses of count data will exceed those presented below.

#### Completion of diagnostic evaluation

In our pilot work, 50% of children who are referred for developmental evaluations complete the evaluation. We aim for a 90% completion rate, which is consistent with our pilot PN study data and assume a marginal improvement in the CCM arm to 65%. With the expected sample, we will have > 90% power to detect clinically significant differences by treatment arm in ITT and explanatory analyses.

#### Time to receipt of recommended services

We assume that 80% in PN arm and 60% in the CCM arm will receive ASD services 5 months after a failed M-CHAT screen. A log rank test will have over 80% power to detect such differences as clinically significant at the 0.05 level.

### Data and safety monitoring board (DSMB)

Our data and safety monitoring board, consisting of autism intervention experts, will meet quarterly to review study procedures. Any adverse events will be reported immediately to both the IRB and DSMB.

### Implementation evaluation

Our evaluation of PN implementation will be done prospectively using a mixed methods approach. Three aims will be carried out sequentially, with each project informing the next. Data will also be triangulated in the final analysis. In aim 1, using a Failure Modes and Effects Analysis, we will lead a team of stakeholders in mapping the navigator intervention to identify and analyze failures in implementation. In aim 2, we will use semi-structured interviews, based on the failures identified in aim 1 and CFIR, to assess barriers and facilitators to implementing navigation. In aim 3, we will use multilevel statistical modeling to examine parent, providers, organizational, and state-level factors that predict implementation success. Success will be defined as fidelity to the intervention, which will be defined as completion of each of the 32 components of the navigation model (e.g. initial family contact, sending introductory letter to PCP, assisting in paperwork for DBP intake visit, completing social security insurance application).

### Process mapping and analysis

Using a Failure Modes and Effects Analysis (FMEA), we will lead a team of stakeholders in mapping navigation to identify and analyze failures in implementation. FMEA is a systematic method for evaluating where intervention processes can fail, and assessing the relative impact of different failures [39]. FMEA follows a proscribed methodology involving process mapping and evaluating failure modes, causes, and consequences. Originally used in engineering fields, FMEA is used regularly in healthcare settings [39], and is particularly useful in evaluating processes – like PN– *prior* to real-world implementation.

We will assemble three multidisciplinary teams that uniquely reflect PN processes in each of the study venues. Participants will include primary care pediatricians, social workers, navigators, nurses, project coordinators, clinic directors, an expert in PN, and an expert in ASD services for minority children. Through a series of face-to-face and videoconference meetings, these multidisciplinary teams will map the process of PN – focusing specifically on screening and identifying participants; engaging participants with their navigators; and delivering navigation with appropriate safety and fidelity.

For each step in the process map, teams will list all possible failure modes (i.e. anything that has gone, or could go, wrong in implementing the step), along with the causes and consequences of that failure. For example, in delivering PN with proper fidelity, a process failure could occur if an insufficient number of sessions are delivered, or if the patient navigator practices the technique incorrectly. Whereas the former – insufficient number of sessions delivered - might be caused by a mismatch in schedules between navigator and parent, the latter – incorrect technique - might be caused by insufficient navigator training or supervision.

#### Risk priority calculation

For each failure mode, we will calculate a risk priority number – a composite score that reflects three fundamental dimensions of an actual or latent failure and the risk that it poses to program implementation: likelihood of ocurrence, likelihood of detection, and severity. Each team will assign a numeric value between 1 and 10 to each dimension; the risk priority number is the product of the three and thus ranges between 1 and 1000. Failure modes with the highest risk priority numbers represent significant threats to implementation and will be probed in depth among participants (aim 2).

#### Comparative analysis across settings

We will compare each of the above indices across study venues – noting similarities and differences of all actual and potential failures. We will compare the acceptability of navigation to families in each venue and determine whether differences in fidelity exist by delivery system*.*

### Qualitative interviews

We will employ CFIR [24] to conduct a series of semi-structured interviews with key stakeholders to evaluate barriers and facilitators to delivering PN. The purpose of these interviews is to better understand the high risk failure modes identified in our process mapping. The component domains of CFIR will guide the interview protocol (Intervention Characteristics, Outer setting, Inner setting, Characteristics of Individuals, Process). Participants will be grouped into the following categories: a) physicians (*n* = 30), clinic administrators and directors (*n* = 8) and policy experts (*n* = 6); b) navigators (n = 8); and c) parents of children with ASD (*n* = 45). We will probe answers to understand how each CFIR construct relates to their experience. Our questions will focus on understanding both the individual perspective and the contextual impact.

We will use content analysis and a deductive approach. We will use the CFIR coding framework (available at www.cfirguide.org/) [32] to code our data, but will also be open to new themes that may emerge. Our coding process will be guided by consensual qualitative methods. Multiple coders will be used throughout data analysis process to foster various perspectives and validation will be carried out through deliberation and consensus. A summary memo will be developed for each category of interviewee using a two-level deliberated consensus approach. First, two investigators will independently code an individual transcript. Then, they will meet and compare coding and agreed on final codes. Finally, they will write a case memo, organized by CFIR construct with summary statements and supporting quotes. Investigators will refine the memo as they continue analysis, until all transcripts are coded, using each new transcript to confirm previous summary statements or add new information. The study team will meet weekly to review memos.

Each memo will be rated using a consensus process to assign a rating to each construct within each category of interviewee (parent, provider, navigator, policy expert). Ratings will reflect the direction of influence (positive or negative) and the magnitude of each construct. Once all constructs for all cases are rated, we will compare ratings for each construct across categories. This approach combines the strengths of a case-oriented method, which allows for rich context-specific consideration when rating each construct, with a variable-oriented method, which promotes identifying patterns and relationships by construct across cases to heighten overall validity of ratings.

### Multilevel model

We will use multilevel statistical modeling to examine parent, navigator, organizational, and state policy factors that predict implementation success (Table 2). Success will be defined as fidelity to the intervention model. Navigator fidelity will be the primary outcome as it a critical component of implementing any intervention, and the most commonly measured implementation metric [32]. We will measure fidelity at the completion of the navigator protocol. Fidelity will be defined as: 1) completion of the three home visits; 2) completion of Navigator log; and 3) adherence to the eight constructs of motivational interviewing expected to be performed by the navigators.

**Table 2:** Measures for Multi-level Model of Navigator Fidelity

Measure	Tools	Level
Provider Density	Publically available data on certified ASD providers	State
Diagnostic Availability	Publically available data on number of ASD diagnostic centers	State
Characteristics	Organization location, size, and type	Organizational
Organizational Readiness to Change	Organizational Readiness to Change Assessment tool [52]	Organizational
Demographics	Age, race, ethnicity, gender, education, language proficiency	Navigator
Experience with ASD	Question developed by study team	Navigator
Cultural competency	Dogra’s Cultural Awareness Questionnaire [53]	Navigator
Navigator proficiency	Measure developed from Navigator training manual	Navigator
Demographics	Age, race, ethnicity, child’s gender, comorbid conditions, parental education, nativity, English proficiency, access to transportation, insurance	Parent
Parental social support	MOS-Social Support Survey [54]	Parent
Global perceived stress	Perceived Stress Scale- Self Report 14 item [55]	Parent
Parenting stress	Parenting Stress Index 4th edition – Short Form [56]	Parent
Physical/emotional health	Veterans RAND 12 Item Health Survey [57]	Parent
Parental coping strategies	Brief COPE [58]	Parent
Child’s impact on the family	Family Impact Questionnaire [59]	Parent
Parenting stress from ASD	Autism Parenting Stress Index [60]	Parent
Parental perceived mastery	Pearlin Mastery Scale [61]	Parent
Child’s adaptive functioning	Adaptive Behavior Assessment System II [62]	Parent

We will combine three data sets: parent-reported measures collected at three time points throughout the study; primary surveys of navigator and clinic data collected in year one (clinic) and three (navigator) of the project; and publically available state-level data.

First, we will evaluate bivariate associations between parent, navigator, organizational, and state-level predictors and navigation model fidelity using χ2 test for pairs of categorical variables and 1-way ANOVA for comparing continuous and categorical variables. Since the data has a 4-level structure, with many individual families nested within navigators, navigators nested within organizations, and organizations nested within states, multilevel logistic regression will be used to account for the lack of independence. We will calculate the intraclass correlation in a null model to analyze how much of the variance in our outcome can be potentially attributed to the predictor level. This will be followed by a series of models: model #1 will examine the unadjusted association between state resources and outcomes, model #2 will add organizational characteristics to model #1; model #3 will add to model #2 by adjusting for navigator level characteristics; model #4 will further adjust model #3 for parent characteristics, and finally, model #5 will add to model #4 by adjusting for parent-reported measures.

In our sample of three states, we propose to evaluate data from 12 clinics, 8 patient navigators and 125 parents. To account for the clustered design, our estimation of power was adjusted via a design effect using an estimate of within subject correlation of the outcome parameter. A proportion of non-fidelity at the mean of 20% compared to a proportion of 0.10 at 1 SD above the mean for the predictor will yield an odds ratio of 0.44. Assuming the design effect will be no larger than 1.41 (the square root of 2), the statistical power should be computed based on a sample size of 89 for an observed sample of 125 parents. For 89 parents, the above-noted difference with an odds ratio of 0.44 will have 82% power with a two-sided alpha of 0.05.

## Discussion

The current study is advances the field of ASD services for multiple reasons. First, it is the first large randomized trial of an intervention specifically designed to improve utilization of evidence-based care for children with ASD. Children with ASD experience significant delays in care, and many engage in alternative, or non-evidence based practice [40]. If proven effective, future work may consider prolonging the intervention to continue to assure appropriate utilization of EBS for this children beyond the initial diagnostic period.

Second, we are testing an intervention designed to alleviate disparities. Addressing disparities is particularly important for children with ASD, who experience significant differences in utilization of EBS based on racial, ethnic, and socioeconomic status [41–44]. Minority families of children with ASD may be particularly vulnerable to disparities in service engagement. For example, a diagnosis of ASD is considered stigmatizing in certain cultures [10], and a fear of stigma may create barriers to both obtaining a diagnosis and engagement in services [45]. Moreover, compared to many other neurodevelopmental conditions, ASD services require intensive parent involvement and training. [46] These requirements may be difficult for families who lack financial resources, support networks, are non-English speaking, or who have other competing demands. Multiple studies show that cultural attitudes towards child development and developmental disabilities shape how minority families respond to the challenges of ASD [45, 47]. studies show Latina mothers to have limited knowledge about autism, and often have differing views of their children’s developmental and the importance of treatment services compared to their medical providers [48]. In their study of South Asian immigrant families [45], Jegatheesan et al. report a perceived gap between service providers’ and families’ views of the child’s home. Whereas providers viewed the home as a stable, closed environment, families emphasized the importance of flexibility and openness to relatives and community members. They observed that differing perspectives resulted in families withdrawing from services. Taken together, these data suggest that testing a family-focused, culturally sensitive intervention designed to address disparities is particularly important in this population.

Finally, given the hybrid effectiveness/implementation design, at the end of the study, we will both know if PN is effective, and have collected data that can inform the rapid, large-scale dissemination of PN for vulnerable children with ASD. These data may also be applicable to those working to disseminate other systems-based strategies designed to improve access to services for children at risk for ASD or other mental health or developmental disorders [24]. Finally, we anticipate these data will provide insight to investigators, community organizations, or states working to implement PN interventions for both ASD and other common disorders that experience disparities in care such as asthma or HIV [15, 49].

## Abbreviations

ASD: Autism spectrum disorder
PN: Patient Navigation
